# Low Cubilin/Myeloperoxidase ratio as a promising biomarker for prognosis of high-grade T1 bladder cancer

**DOI:** 10.1007/s11255-024-03971-4

**Published:** 2024-03-26

**Authors:** Mariana Silva Medeiros, Luís André Botelho de Carvalho, Marta Alves, Ana Papoila, Hugo Miguel Baptista Carreira dos Santos, José-Luis Capelo-Martínez, Luís Manuel Viegas de Campos Pinheiro

**Affiliations:** 1 Department of Urology, Central Lisbon University Hospital Centre, Lisbon, Portugal; 2 Department of Chemistry, Faculty of Science and Technology, Caparica, Portugal; 3 Epidemiology and Statistics Unit, Research Centre, Central Lisbon University Hospital Centre, Lisbon, Portugal

**Keywords:** Urinary proteome, Recurrence, CUBN/MPO, Non-muscle-invasive bladder cancer

## Abstract

**Purpose:**

T1 bladder cancer is known for its high progression and recurrence rates. Identifying aggressive tumours at the non-muscle-invasive stage is crucial to allow early interventions and subsequently increase patient survival. This study aimed to investigate the potential of the cubilin/myeloperoxidase (CUBN/MPO) ratio as a high-grade T1 bladder cancer biomarker.

**Methods:**

Urine samples were collected from 30 patients who underwent transurethral resection of the tumour with high-grade T1 bladder cancer (June 2015 to December 2019) before surgery. The urinary proteome was analysed using high-resolution mass spectrometry and the CUBN/MPO ratio was calculated. The primary outcome was the recurrence during the follow-up (around 31.5 months after resection). Univariate Cox regression and Kaplan–Meier curves were used for data analysis.

**Results:**

Patients with a low CUBN/MPO ratio exhibited upregulated MPO and/or downregulated CUBN. This group of patients had a higher incidence of disease recurrence and progression. Low CUBN/MPO ratio was significantly associated with a higher likelihood of recurrence, progression, and death. It is worth noting that this study was exploratory and conducted on a small sample size, so further research is needed to validate these findings in larger cohorts.

**Conclusion:**

This study highlights the potential of the CUBN/MPO ratio as a prognostic biomarker for high-grade T1 bladder cancer.

**Supplementary Information:**

The online version contains supplementary material available at 10.1007/s11255-024-03971-4.

## Introduction

Stage T1 bladder cancer has the highest progression and recurrence rate of non-muscle-invasive bladder cancers (NMIBCs) [1]. T1 bladder cancer is a heterogeneous disease, with patients showing an almost indolent illness contrasting with high number of patients dying due to this disease. Some patients who experience disease progression to the muscle-invasive stage have a worse prognosis than those who have ‘primary’ muscle-invasive disease [2, 3]. Identifying these more aggressive tumours when they are still non-muscle-invasive, is imperative to offer early cystectomy and save lives [3]. However, it is not possible to easily identify this subgroup of high-grade (HG) stage T1 bladder cancer and identify it early due to the lack of biomarkers [4].

Proteome analysis has been used to identify new promising biomarkers to detect and to make an accurate prognosis of bladder cancer [5, 6]. Urine is in direct contact with bladder tumour and this makes the urinary proteome a “gold clinical source”, allowing to search for proteins that may be used as biomarkers due to proteins having a close connection with the phenotype with a not invasive way [5]. Previously, it was demonstrated that a panel of urinary proteins measured by high-resolution mass spectrometry could predict bladder cancer stages, namely, Ta, T1 and T2 cases [7]. Moreover, most studies evaluate all stages of bladder cancer (BC) and very few of them found urinary biomarkers that could subgroup the T1 BC adding additional prognostic accuracy to what is already known [4].

Performing longitudinal profiling with individual proteomes has been described in the literature as a possibility of following the course of non-muscle-invasive bladder cancer, using high-resolution mass spectrometry. In this paper, we have conscientiously explored the extensive raw data from our earlier work by Carvalho et al. [8]. This complex dataset collection was collected through considerable effort and enabled diverse analyses. Previously, we developed a novel approach for monitoring bladder cancer progression based on a differential personal pathway index (dPPi), which uses variations in the expression of specific biochemical pathways to indicate disease progression and the potential need for medical intervention. In contrast, this paper investigates a specific biomarker, the CUBN/MPO ratio, in high-grade T1 bladder cancer. We verified that repurposing this data in such a manner is an efficient and responsible use of resources, circumventing the need for redundant data acquisition [8, 9].

Myeloperoxidase (MPO) is a haeme protein synthesized during myeloid differentiation that constitutes the major component of neutrophil azurophilic granules. MPO gene has been mapped to the chromosomal region 17q22 [10]. The main known function of this enzyme is its microbicidal activity due to hypohalous acid production [11]. Furthermore, MPO has been described as a key enzyme in carcinogenesis, as well as in the progression of cancer [12].

Cubilin, the receptor for intrinsic factor vitamin B12, is a 460-kDa endocytic receptor co-expressed with megalin [13]. This protein has functions in vitamin B12 homeostasis and renal reabsorption of protein or toxic substances including albumin, vitamin D-binding protein or cadmium [14]. It plays an important role in the development of the peri-implantation embryo through internalization of apolipoprotein-I (APOA1) and cholesterol [15, 16]. The direct influence of this protein in pathways involved in cancer is unknown.

In this study, we hypothesized that the ratio of CUBN/MPO can play an essential role in identifying patients who experience a poorer prognosis. We aimed to evaluate if the low ratio of CUBN/MPO in urine has a prognostic value on HG T1-staged patients.

## Materials and methods

### Urine collection and patient follow-up

T1 HG bladder cancer was classified according to the TNM classification system approved by the Union International Contre le Cancer (UICC), which denotes tumour invasion into the subepithelial connective tissue. The grade was determined based on The WHO 2002 classification system [17].

Mid-stream second void morning urine samples of 30 patients who underwent transurethral resection of a bladder tumour (TURB) from June 2015 to December 2019 were collected. The urinary proteome from the HG pT1 patients randomly selected was analysed using high-resolution mass spectrometry. Patients were informed about the study and provided informed consent according to the policies of the Central Lisbon Hospital Centre Ethics committee (Number 669/2018). Additionally, patients were followed and underwent regular clinical evaluation according to EAU guidelines to diagnose recurrences and progression to muscle-invasive tumours.

### Urine sample treatment

Urine samples were collected in centrifuge tubes containing 38 mg of boric acid to prevent bacterial growth [18]. Samples were centrifuged at 5000*g* for 20 min to remove cell debris. The resulting supernatant was then divided into 10 mL aliquots and stored at − 60 °C. Prior to analysis, a 10 mL aliquot was thawed and concentrated to a volume of 300 µL using a VivaSpin 15R (cutoff 10 kDa) through centrifugation at 5000*g* for 20 min. The total protein content of samples was then quantified using the Bradford method assay.

### Filter-aided sample preparation (FASP) and LC–MS/MS

A total amount of 100 µg of protein and two technical replicates of each urine sample were processed using the filter-aided sample preparation (FASP) method, which has been developed for rapid urine proteome isolation, cleaning, and digestion [19]. Briefly, urine proteome was loaded onto a 10 kDa molecular weight cutoff FASP membrane and cleaned using a solution of 8 M urea 25 mM ammonium bicarbonate (AmBic). Furthermore, proteins were reduced and alkylated using, respectively, 50 mM dithiothreitol (DTT) and 50 mM iodoacetamide prepared in 8 M urea and 25 mM AmBic solution. Following that, protein digestion was carried out overnight (16 h) at 37 °C and the resulting peptides were collected, dried and stored at − 20 °C until further analysis. Finally, peptides were resuspended in 200 µL of 3% (v/v) acetonitrile containing 0.1% (v/v) aqueous formic acid and the total amount of peptides was quantified by a colorimetric pierce™ quantitative colorimetric peptide assay. The peptides were subjected to high-resolution LC–MS/MS using an Ultimate 3000 nano LC system coupled to an Impact HD (Bruker Daltonics) with a CaptiveSpray nanoBooster.

### Bioinformatics

Protein identification and label-free quantification (LFQ) were achieved by using the MaxQuant V1.6.0.16 software with the integrated peptide search engine Andromeda. The processing of all raw data files occurred in a unified batch using the standard settings recommended by the software [20]. For database querying, the Andromeda search engine was used, referencing the UniProt database (UP000005640_9606), which contains 20,600 sequences and 11,395,157 residues (as of April 27, 2021). Perseus software (version 1.6.15.0) was employed for data processing [21].

Protein group LFQ intensities was transformed using a log2 scale and the data filtered to exclude missing values (minimum valid percentage of 50% in at least one group and values > 0). To address the issue of missing LFQ values, imputation was performed from the overall matrix (width of 0.5 and a downshift of 1.8) [19]. The log ratios were derived from the average log2 LFQ intensity differences between conditions, using a two-tailed Student’s *t* test with a permutation-based FDR of 0.05 and an S0 of 0.1.

### Statistical analysis

IBM SPSS Statistics Version 25 was used to prepare the graphs and perform the statistical analysis, including the estimation of Kaplan–Meier survival curves, the use of log-rank tests for comparing survival functions, and the use of Cox regression analysis to obtain crude hazard ratio (HR) estimates. Since there is no normal cutoff for CUBN/MPO in urine described in literature, we used martingale residuals to obtain a cutoff point. The CUBN/MPO ratio influences on overall survival, recurrence and progression rate was analysed considering this variable as continuous and as binary (after discretization using the obtained cutoff point). Non-parametric Fisher’s exact and Mann–Whitney tests were applied, as appropriate.

A level of significance *α* = 0.05 was considered.

### Study definitions and outcomes

For the analysis of results in this study, the following definitions were considered:

Recurrence: reappearance of non-muscle-invasive bladder cancer during follow-up verified by pathological examination, after complete excision or the second TURBT.

Progression: appearance of muscle-invasive bladder cancer or radiological evidence of locally advanced bladder tumour.

Overall survival: time since the first TURBT until the date of death or follow-up.

The follow-up included progression-free survival, disease-specific survival, and overall survival, and measured from the time of primary surgery to the time of the proven event (recurrence, progression or death).

Patients were considered BCG refractory under the following conditions: if T1 HG/G3 tumour is present at 3 months; if Ta HG/G3 tumour is present after 3 months and/or at 6 months, after either re-induction or first course of maintenance of BCG; if HG tumour appears during BCG maintenance therapy and whenever an MIBC is detected during follow-up.

## Results

### Patient sample characterization

The sample included a total of 30 patients, 29 male and 1 female patient, with mean age of 72 years (sample characteristics are summarized in Table 1). The median follow-up time for the 30 patients analysed in this study was 26.50 months, ranging from 6 to 75 months. All the patients had T1 HG bladder cancer (Table 1) and none presented CIS (carcinoma in situ).

**Table 1:** Patient characteristics based on the CUBN/MPO ratio

	Total	Low CUBN/MPO ratio	High CUBN/MPO ratio	*p* value
*N*	30	7	23	
Follow-up in months median (*P*_25_; *P*_75_)	26.5 (10.75–51.0)	24.0 (9.0–31.0)	27.0 (11.0–65.0)	0.413**
Mean age in years (SD)	72 (1.64)	76.29 (3.39)	70.70 (1.82)	0.245**
Number of lesions				0.399*
One	14/30 (46.7%)	2/7 (28.6%)	12/23 (52.2%)	
Multiple	16/30 (53.3%)	5/7 (71.4%)	11/23 (47.8)	
Appearance				0.154*
Pappilary	23/30 (76.7%)	7/7 (100%)	16/23 (69.6%)	
Sessile	7/30 (23.3%)	0/7 (0%)	7 /23 (30.4%)	
Size				0.143*
< 3 cm	8/30 (26.7%)	0/7 (0%)	8/23 (34.8%)	
≥ 3 cm	22/30 (73.3%)	7/7 (100%)	15/23 (68.2%)	
Incomplete TURB	5/30 (16.7%)	2/7 (28.57%)	3/23 (13%)	0.565*
Second TURB	16/30 (53.3%)	4/7 (57.1%)	12/23 (52.2%)	1.000*
T1–T2 on second TURB				0.547*
Yes	5/16 (31.3%)	2/4 (50%)	3/12 (25%)	
No	11/16 (68.8%)	2/4 (50%)	9/12 (75%)	
BCG/adjuvant MMC				0.603*
Yes	24/30 (80%)	5/7 (71.4%)	19 (82.6%)	
No	6/30 (20%)	2/7 (28.6%)	4 (17.4%)	
BCG refractory				1.000*
Yes	9/22 (40.9%)	1/5 (20%)	5/17 (29.4%)	
No	13/22 (59.1%)	4/5 (80%)	12/17 (70.6%)	
Recurrence-free survival median (95% CI) months	39 (0.00–82.92)	5 (0.00–10.13)	69 (23.45–114.44)	< 0.001^#^
Progression-free survival median (95% CI) months	^##^	16 (0.79–31.21)	^##^	0.004^#^
Overall survival median (95% CI) months	^##^	26 (13.73–38.27)	^##^	0.005^#^

*CI* confidence interval, *SD* standard deviation

*Fisher’s exact test; **Mann–Whitney test; ^#^Log-rank test; no median estimate was obtained because more than half of the patients were still living

Concerning the number of tumours, the majority of patients (53.3%) had more than one, and the percentage was higher in the group with a lower ratio (71.4%). Tumours were over 3 cm in most cases, and all patients with a low ratio had tumours larger than 3 cm.

Five patients (16.7%) underwent an incomplete first TURB, and four patients (13.3%) did not have any detrusor in the specimen of TURB. In these five cases, the lesions were very extensive and the initial TURB was insufficient for complete excision. In these cases, a second TURB was performed to finalize the resection, and recurrence was defined as the appearance of a tumour after the second TURB.

Only one patient did not complete the bladder cancer excision due to imaging progression after 2 months. The patient was 91 years old and had an incomplete TURB due to extensive urothelial carcinoma with muscular mucosa present in the specimen without carcinoma; however, imaging progression was noted 2 months after the initial surgery. As the patient was not fit for curative surgery, they continued palliative care and survived for 24 months.

Although all patients should have undergone a second TURB, half of them did not undergo this procedure because they presented withHG pT1 bladder cancer. The study conducted was retrospective, and undergoing a second TURB was not an inclusion criterion. Another patient refused the second TURB, received adjuvant BCG therapy and remained free of disease recurrence during a 28-month follow-up. For the remaining 12 patients, no reasons were found for not undergoing the second TURB.

A second resection was performed for better stage definition in 16 patients (53.3%) and 9 of these had residual tumour: 3 patients with pTa, 4 pT1, 1 pT2 and 1 CIS. Twenty-two patients (73.3%) were treated with BCG (30% with induction BCG and 43.3% with induction and maintenance BCG) and 2 patients with adjuvant MMC (mitomycin C) instillations (6.7%). Unfortunately, there was a stock shortage of BCG, leading these two patients to undergo mitomycin treatment.

### Hierarchical clustering analysis of MPO and CUBN patients’ expression levels

**Fig. 1:**
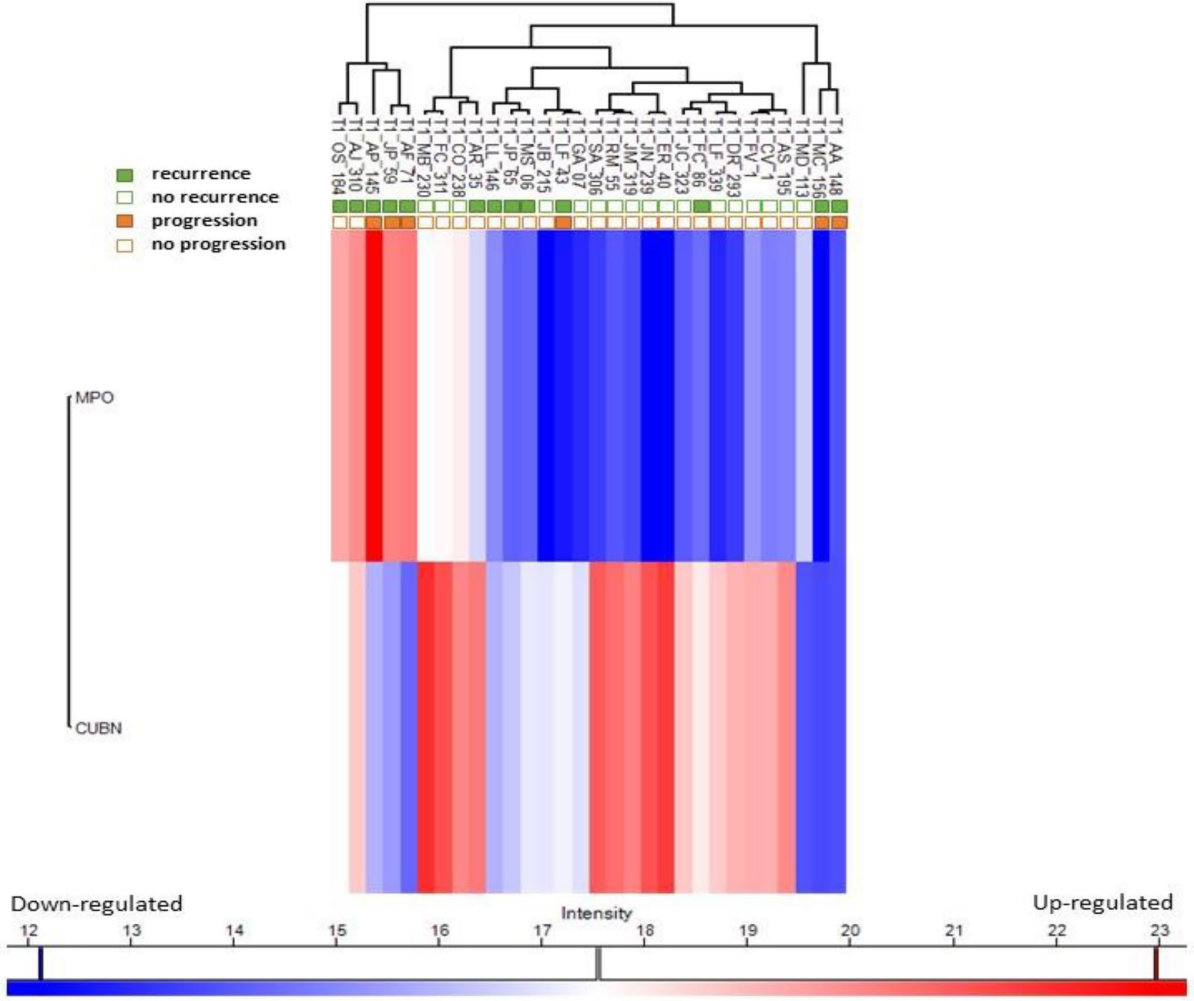
Hierarchical clustering analysis of MPO and CUBN patients’ expression levels as well as the occurrence of tumour recurrence or progression throughout follow-up

Protein myeloperoxidase (MPO) was found to exhibit the greatest increases in levels in patients with recurrence and progression (the first 5 patients on the left) (Fig. 1). Conversely, the protein cubilin (CUBN) presented higher levels in patients without evidence of disease progression or recurrence.

The analysis of the data in Fig. 1 shows a distinct pattern among the patients, and three main clusters were observed. In the first cluster, the first five patients showed an upregulation of MPO coupled with a downregulation of CUBN (cubilin), resulting in a low ratio. This group of patients exhibited recurrence, with three of them experiencing disease progression. In the second cluster, 22 patients stood out as they exhibited downregulated levels of CUBN (19 out of 22), resulting in a higher CUB/MPO ratio. Interestingly, among these patients, only one individual (LF-43) displayed disease progression, while 21 of 22 did not experience further progression. Finally, a third cluster was identified, characterized by the downregulation of both MPO and CUBN levels. Within this cluster, three patients were observed, two of whom experienced both recurrence and disease progression (MC-156 and AA-148).

Therefore, lower ratio value of CUBN/MPO was shown to be related to upregulated MPO and/or downregulated CUBN (Fig. 1), as well as, interestingly, lower ratio value seems to be associated with disease recurrence and progression. In fact, the percentage of patients with recurrence and progression was superior in the subgroup of low-value CUBN/MPO ratio (Table 1).

### Prognostic value of the CUBN/MPO ratio

After finding that the expression of MPO was associated with an increased risk of recurrence and progression, and in contrast, the expression of CUBN was associated with a lower risk of recurrence and progression (Tables 2 and 3) in univariable Cox regression, we evaluated the CUBN/MPO ratio to achieve greater prognostic accuracy by incorporating both markers into a single variable.

**Table 2:** Univariable Cox regression analysis for recurrence-free survival

	Hazard ratio (95% CI)	*p* value
Age (per 5-year increment)	1.357 (0.881–2.075)	0.167
Gender (female)	0.186 (0.022–1.591)	0.124
Appearance/sessile	1.566 (0.337 – 7.276)	0.567
Number/multiple	1.487 (0.480–4.606)	0.491
Size (≥ 3 cm)	6.261 (0.806–48.658)	0.080
Complete TURB	0.568 (0.165 – 1.958)	0.370
Muscular mucosa present in specimen	1.002 (0.206–4.872)	0.998
Recurrence rate (recurrent)	0.943 (0.283–3.141)	0.924
Second TURB	0.848 (0.282–2.549)	0.769
Time to the second TURB (per 7-days increment)	0.679 (0.425–1.083)	0.104
Presence of tumour in the second TURB	3.708 (0.433–31.795)	0.232
Adjuvant therapy	1.756 (0.380–8.114)	0.471
CUBN	0.700 (0.555–0.883)	0.003
MPO	1.279 (1.067–1.534)	0.008
CUBN/MPO ratio	0.007 (0.001–0.095)	< 0.001
Low CUBN ratio	12.963 (2.808–59.841)	0.001

The reference categories were *male* for “Gender”, *papillary* for “Appearance”, *one* for “Number”, *less than 3 cm* for “Size”, *No* for “Complete TURB”, *No* for “Muscular mucosa present in specimen”, *No* for the “Presence of tumour in the second TURB”, *primary* for the “Recurrence rate”, *No* for “Adjuvant therapy”, *and high CUBN/ratio* for “CUBN/MPO ratio”

**Table 3:** Univariable Cox regression analysis for progression-free survival

	Hazard ratio (95% CI)	*p* value
Age (per 5-year increment)	1.169 (0.652–2.096)	0.591
Gender (female)	21.677 (0.00–436,032,183.4)	0.720
Appearance (sessile)	0.486 (0.056–4.184)	0.511
Number (multiple)	6.165 (0.715–53.164)	0.098
Size (≥ 3 cm)	32.884 (0.015–70,168.161)	0.372
Complete TURB (yes)	0.202 (0.040–1.008)	0.051
Muscular mucosa present in specimen (yes)	0.894 (0.104–7.674)	0.919
Recurrence rate (recurrent)	0.964 (0.176–5.269)	0.966
Second TURB (yes)	1.635 (0.299–8.939)	0.570
Time to the second TURB (per 7-day increment)	0.661 (0.343–1.274)	0.216
Presence of tumour in the second TURB (yes)	46.859 (0.012–184,320.303)	0.362
Adjuvant therapy (yes)	0.476 (0.087–2.616)	0.393
CUBN	0.534 (0.332–0.857)	0.009
MPO	1.180 (0.939–1.483)	0.156
CUBN/MPO ratio	0.015 (0.001–0.422)	< 0.014
CUBN/MPO ratio (low)	8.008 (1.460–43.942)	0.017

The reference categories were *male* for “Gender”, *papillary* for “Appearance”, *one* for “Number”, *less than 3 cm* for “Size”, *No* for “Complete TURB”, *No* for “Muscular mucosa present in specimen”, *No* for the “Presence of tumour in the second TURB”, *primary* for the “Recurrence rate”, *No* for “Adjuvant therapy”, and *high CUBN/ratio* for “CUBN/MPO ratio”

The univariable Cox regression (Table 2) shows that the risk of having recurrence was approximately 13 times higher in people with low ratio of CUBN/MPO (HR = 12.963, 95% CI [2.808; 59.841]).

The variables age, number, size and tumour appearance, completeness of TURB, presence of muscular mucosa in the specimen, the recurrence rate, time to second TURB and adjuvant therapy were assessed with univariable Cox regression, and they did not demonstrate a statistically significant risk association for recurrence or disease progression (Tables 2 and 3).

In the same way, the risk of having progression was approximately eight times higher in people with low ratio of CUBN/MPO (HR = 8.008, 95% IC [1.460–43.942]).

**Fig. 2:**
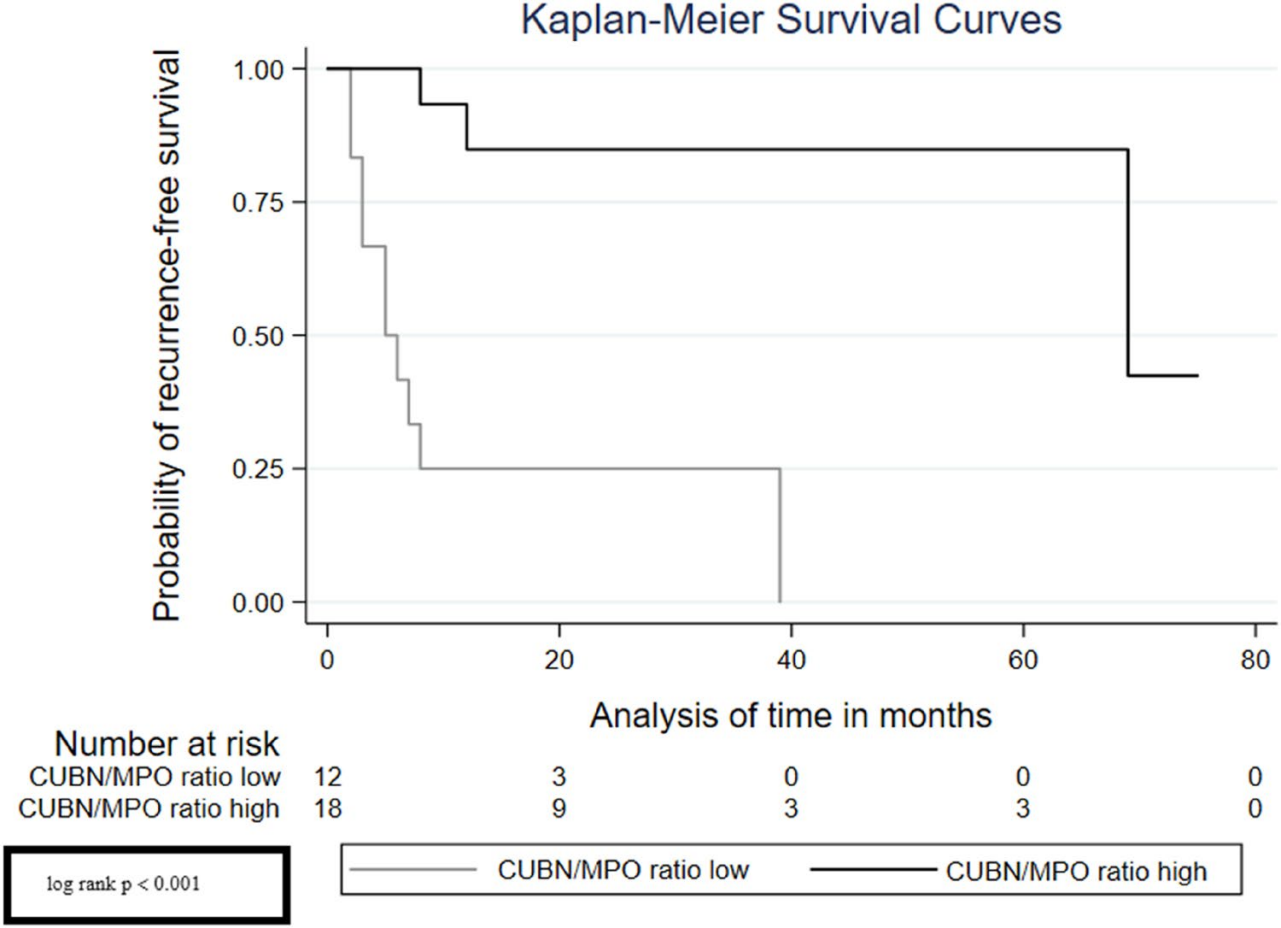
Survival analysis—Kaplan–Meier curves displaying the association of CUBN/MPO ratio with recurrence-free survival

**Fig. 3:**
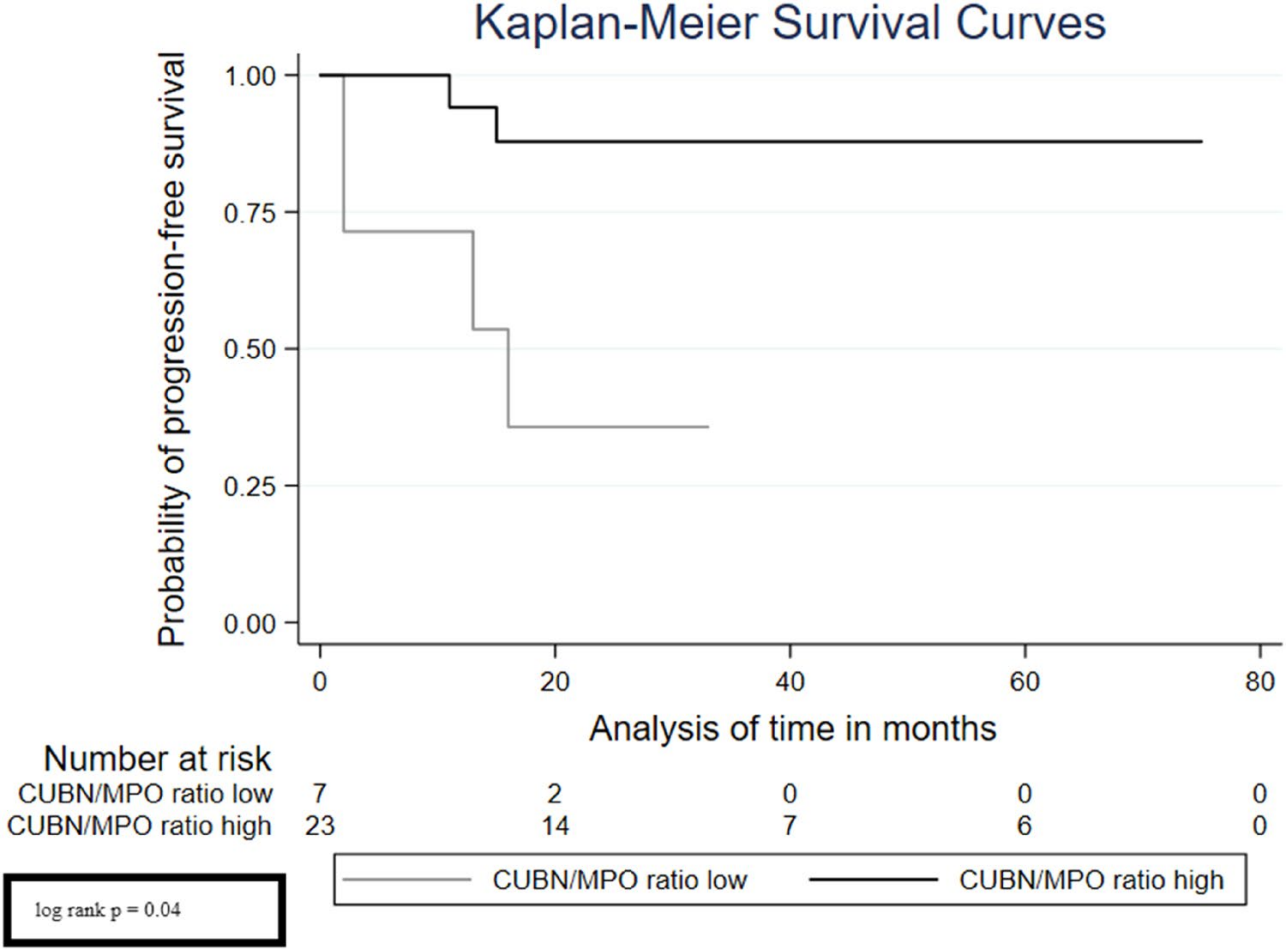
Survival analysis—Kaplan–Meier curves displaying the association of CUBN/MPO ratio with progression-free survival

**Fig. 4:**
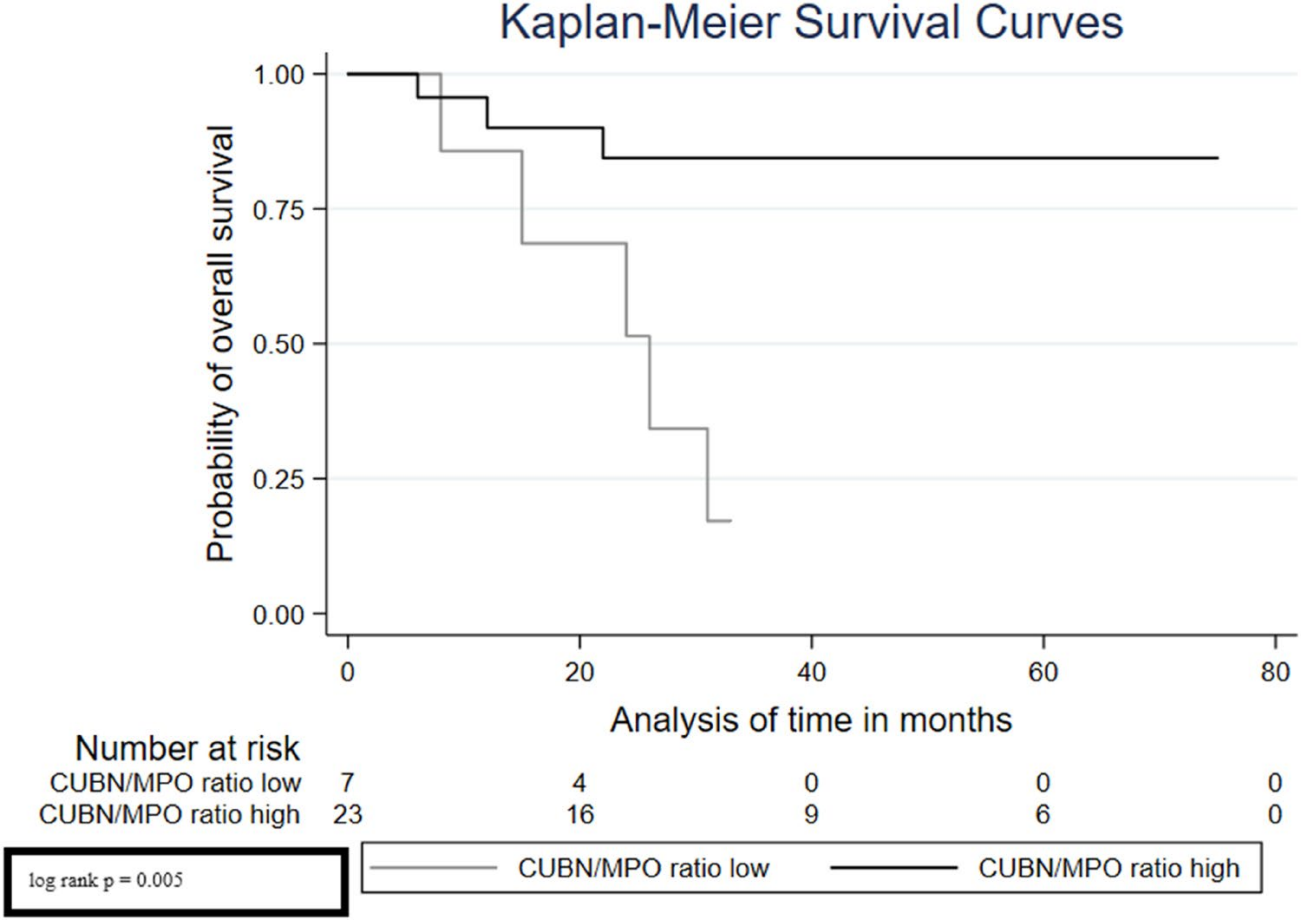
Survival analysis—Kaplan–Meier curves displaying the association of CUBN/MPO ratio with overall survival

Considering the Kaplan–Meier curves, we can see that the low CUBN/MPO ratio was significantly associated with a high risk of recurrence (Fig. 2, log rank *p* < 0.001), progression (Fig. 3, log rank *p* = 0.040) and also a poor overall survival (Fig. 4, log rank *p* = 0.005).

## Discussion

Few studies have explored the ability of urinary biomarkers in predicting the progression of high risk NMIBC to a muscle-invasive state.

The evaluation of the Urovision^®^ Test before BCG treatment failed to differentiate patients who later progressed from those who did not. [22] Kamat et al. found a notable disease progression variance in High Risk NMIBC patients with a positive Urovision^®^ Test (19.8% vs. 4.4%), lacking reliability for clinical use [23]. Piaton et al. in 2014 highlighted using p16INK4a and Ki-67, showing 100% progression likelihood in pTa/pT1 HG BC patients with both markers. Despite this, the test’s sensitivity and specificity did not exceed traditional cytology, making it unsuitable as a clinical substitute [4].

As far as we know, the CUBN/MPO ratio has never been previously studied as a promising biomarker or prognostic factor for urinary cancer or any other type of cancer. However, cubilin (CUBN) has been identified and validated as a marker capable of classifying renal cell carcinoma (RCC) patients into low- and high-risk groups. Loss of CUBN expression has been significantly and independently correlated with less favourable patient outcomes concerning overall survival. Conversely, stratification of patients according to CUBN positivity showed a significant benefit for patients with CUBN-positive tumours in overall survival and RCC-specific survival [16].

Although these results apply to RCC, they point in the same direction as our findings, suggesting that the loss of expression of this protein is associated with a poorer outcome. According to our results, patients with upregulated cubilin did not show disease progression, apart from only one patient who had recurrence. On the contrary, of the patients who exhibited downregulated cubilin (eight patients), seven had recurrence and five had disease progression.

On the other hand, myeloperoxidase (MPO) can support a hypermutagenic environment through the action of MPO-derived oxidants that are able to oxidize and modify DNA. Furthermore, MPO has been involved in pathways of apoptosis, cell migration, tumour growth and adaptive immunity in cancer [12]. Also, it is already known that MPO interferes in the activation of procarcinogens, included in tobacco smoke, such as polycyclic aromatic carcinogens, aromatics amines, heterocyclic amines and the endogenous formation of carcinogenic free radicals [24]. The variant genotypes of MPO may be related to low enzyme activity and polymorphisms in MPO that was related to a decreased risk of bladder cancer developing in Tunisian population [25].

This finding emphasizes the potential importance of MPO in BC carcinogenesis.

Although the protein has not been validated as a severity biomarker in bladder cancer, Valadez-Cosmes et al. showed that MPO expression was associated with lower survival in non-small cell lung cancer patients [26]. Indeed, in our study, the results indicate that patients with downregulated MPO in urine tend to have a better outcome during follow-up, with lower rates of recurrence and disease progression and patients with upregulated MPO tend to have shorter overall survival.

The value in ratio use for both proteins as a biomarker allowed to add discriminatory value in patient selection, mostly in those who had one of the proteins excessively down- or upregulated. Here, we demonstrated that the mean ratio value of CUBN/MPO was significantly lower if patients had experienced bladder tumour recurrence or progression. This result suggests that the lower the CUBN/MPO ratio, the worse will be the prognosis.

In our study, the results showed that the risk of having recurrence and progression was respectively and approximately 13 and 8 times higher in patients with low ratio of CUBN/MPO compared to patients with high ratio of CUBN/MPO. In the univariable Cox regression analysis, it was not possible to demonstrate a statistically significant risk association for recurrence-free survival or progression-free survival for other clinically validated variables known as prognostic factors, such as tumour size and the number of tumours. These results were likely due to the small size of the patient cohort studied, which precluded performing a multivariable analysis. Therefore, this work must be viewed as an exploratory and preliminary study that will need validation in a larger patient population.

An important limitation of this study was the lack of a representative sample of female patients. Only one woman was included in the study. This could have been the reason why the female gender showed a discrepancy in the risk of recurrence-free survival (HR 0.186 (0.022–1.591)) compared to that of progression-free survival 21.677 (0.00–436,032,183.4). A more representative number of both genders should be analysed in further studies, also to allow evaluate possible sex-dependent results. Importantly, age may also play a factor, in that the low CUBN/MPO ratio group was older compared to the high ratio group.

Another limitation of the study, as previously mentioned, was that not all patients underwent a complete initial TURB. Nonetheless, from the interpretation of the survival analysis, it appears that the risk of recurrence was lower in patients who had a complete TURB compared to the group of patients in whom this did not occur. However, it was not statistically significant (*p* = 0.370).

Regarding the analysis for progression-free survival, the *p* value for the complete TURB variable almost reached the threshold of statistical significance (HR 0.202 (0.040–1.008), *p* = 0.051), suggesting that the group of patients with low CUBN/MPO may have been impacted in disease progression due to presenting more extensive tumours, as the size of tumour was so large that complete resection was not achieved in the initial surgery.

However, it is necessary to reinforce that two of the patients with incomplete TURB belonged to the low CUBN/MPO ratio group (28.6%) and three patients to the high CUBN/MPO group (13%), which represents a not very disparate percentage between the groups.

Additionally, the fact that several patients did not undergo a second TURB or showed no evidence of muscle in the first TURB sample raises suspicions that some of these patients may have been sub-staged. However, upon analysing Table 1, it is observed that in the group of patients with a low CUBN/MPO ratio, the proportion of patients who underwent a second TURB is similar to the proportion found in the high CUBN/MPO ratio group, 57.1% and 52.2%, respectively. Also, in the univariable Cox regression analysis, the performance of the second TURB did not show statistical significance for recurrence-free survival and progression-free survival, despite maintaining a beneficial trend associated with lower risk.

Contrary to what the study by Baltaci et al. showed, in our study, an increase in the time between the first and second TURB appears to exhibit a trend towards a lower risk of recurrence and progression. This may be because our study included patients with an incomplete first TURB, which in some way influenced the timing of the second TURB [27]. However, the reasons for the extended duration of the second TURB were not evaluated, which is another limitation.

Proteome analysis plays a key role in the understanding of the carcinogenesis involved in bladder carcinoma. The use of urinary proteins as potential diagnostic biomarkers has been investigated for years [5]. However, this is the first time a ratio between a supposed protective protein (cubilin) and a cancer aggressiveness promoter (MPO) measured in the urine of patients has been studied as a prognostic risk factor to predict recurrence and progression to muscle-invasive T1 bladder tumours.

Years ago, it was considered standard to follow NMIBC patients until muscle invasion occurs and then perform radical cystectomy. Nowadays, we know that some high-grade T1 bladder tumours may progress to aggressive muscle-invasive tumours, and this is considered a deleterious prognostic as they are at serious risk of having systemic nonclinical disease at the time of cystectomy. Also, when there is progression to muscle-invasive bladder cancer (MIBC), the delay of cystectomy has been associated with lower survival [2, 3]. However, there is a lack of tumour prognosis biomarkers that can help clinicians in the selection of those patients who will have progression in the future and intervene with more aggressive treatments before the actual development of muscle invasion. This may improve the decision in individualized treatment.

A study investigated Oncuria™, an assay for diagnosing, predicting, and monitoring BCa response to intravesical BCG treatment. This test evaluated intermediate- to high-risk NMIBC patients before BCG treatment. After BCG therapy, BC recurrence was associated with elevated levels of specific proteins (ANG, APOE, A1AT, CA9, MMP9, MMP10, PAI1, SDC1, VEGFA) in the Oncuria™ panel, demonstrating its potential for assessing the effectiveness of intravesical BCG treatment and predicting relapse risk in BC patients [22].

Shariat et al. developed a tool that combines NMP22^®^ with age, gender and cytology to predict recurrences in specific bladder conditions (pTa G3, pT1 and CIS) with high accuracy. Despite its reliability, this model is not widely used in clinical practice, possibly due to uncertainties about its threshold for positivity, adaptability across institutions and a relatively high rate of false positives [28].

Despite the efforts to identify precise biomarkers predicting oncologic results in high-risk NMIBC, conflicting or ambiguous findings hinder the incorporation of these supplementary methods into clinical practice.

Our results suggest that low CUBN/MPO ratio could be a biomarker to select aggressive high-grade T1 bladder cancer patients to be candidates to early cystectomy and improve their oncologic outcome; however, a prospective trial will be necessary. This retrospective study has many limitations including the low number of patients, the inclusion of patients with incomplete first TURB and with no detrusor in specimen and patients that did not undergo a second resection or treatment with BCG. However, despite these limitations, these results must be considered as a preliminary work and further work should be conducted to evaluate and validate the role of CUBN/MPO ratio and to find the biological mechanisms underlying the relationship between CUBN and MPO in high-grade T1 bladder cancer.

### Supplementary Information

Below is the link to the electronic supplementary material.

Supplementary file1 (XLSX 15 KB)

## Data Availability

The data that support the findings of this study are available on request from the corresponding author.
